# Short-term effects of alcohol detoxification on cardiovascular, hematological, and oxidative stress biomarkers: A prospective cohort study

**DOI:** 10.1371/journal.pone.0352164

**Published:** 2026-07-02

**Authors:** Ashraf Mahmoud Emara, Anoud Maseer Ateeq Alshammari, Ahmad H. Alhowail, Rehab Mohamed Elgharabawy

**Affiliations:** 1 Department of Pharmacology and Toxicology, College of Pharmacy, Qassim University, Saudi Arabia; 2 Department of Pharmacology and Toxicology, Faculty of Pharmacy, Tanta University, Tanta, Egypt; University of Diyala College of Medicine, IRAQ

## Abstract

**Introduction:**

Chronic alcohol consumption induces multisystem dysfunction, including cardiovascular instability, hematological alterations, and oxidative stress imbalance. However, the extent of short-term recovery following structured detoxification remains incompletely characterized.

**Objective:**

To evaluate the short-term effects of a standardized inpatient alcohol detoxification protocol on cardiovascular, hematological, and oxidative stress biomarkers.

**Methods:**

A prospective observational before-and-after cohort study was conducted on 75 male participants, including 50 patients with alcohol use disorder and 25 healthy controls. Patients underwent a 21-day inpatient detoxification program comprising pharmacological stabilization and nutritional rehabilitation. Clinical and biochemical parameters—including body mass index, vital signs, lipid profile, complete blood counts, troponin-I, and oxidative stress markers (TBARS, glutathione peroxidase, and catalase)—were assessed at baseline and post-treatment.

**Results:**

Compared to healthy controls, treatment-naïve patients with alcohol use disorder (AUD) exhibited significantly elevated baseline levels of lymphocytes, total cholesterol, low-density lipoprotein (LDL), thiobarbituric acid reactive substances (TBARS), and catalase (CAT), alongside significantly lower heart rate (HR), systolic blood pressure (SBP), neutrophil-to-lymphocyte ratio (NLR), high-density lipoprotein (HDL), and glutathione peroxidase (GPx). Baseline anthropometric, vital, hematological, and metabolic markers—including BMI, diastolic blood pressure (DBP), hemoglobin (Hb), platelets, total white blood cell count (WBCs), cardiac troponin-I (cTnI), and triglycerides—showed no statistically significant differences. Following a 21-day inpatient detoxification protocol, post-treatment assessments revealed significant increases in red blood cell parameters (RBCs, Hb, Hct), neutrophils, lymphocytes, NLR, and HDL. Conversely, standard detoxification induced significant reductions in cardiovascular, lipid, and oxidative stress indices, specifically HR, SBP, mean corpuscular volume (MCV), total WBCs, cTnI, total cholesterol, triglycerides, TBARS, and CAT. No significant post-treatment alterations were observed in BMI, DBP, platelet counts, LDL, or GPx.

**Conclusion:**

Short-term alcohol detoxification leads to partial recovery of cardiovascular and hematological parameters, while oxidative stress markers show limited normalization within the 21-day period. These findings highlight differential recovery patterns across biological systems following early abstinence.

## Introduction

Chronic alcohol consumption is a primary driver of systemic multi-organ pathology, mediated largely by the toxic effects of acetaldehyde and the overproduction of reactive oxygen species (ROS) [1–3]. This imbalance between ROS generation and the endogenous antioxidant defense system results in profound oxidative stress, leading to the deleterious peroxidation of lipids, proteins, and DNA across various tissues [4,5]. While the liver remains a primary site of ethanol metabolism, there is unambiguous evidence that extra-hepatic systems—including the cardiovascular, hematological, and renal systems—undergo significant functional deterioration through both oxidative and non-oxidative pathways [6–8].

At the clinical level, chronic alcohol abuse precipitates a state of ‘metabolic debt,’ characterized by altered energy homeostasis, reduced Body Mass Index (BMI), and dysregulated lipid metabolism [9,10]. Furthermore, alcohol-induced oxidative stress frequently manifests as hematological impairment, typically presenting as macrocytic shifts in mean corpuscular volume (MCV) and reduced hemoglobin levels [9,11]. Despite the established risks of cardiac depression and secondary cardiomyopathy associated with these metabolic disruptions, there is a significant gap in the literature regarding the longitudinal recovery of these biomarkers following standardized clinical intervention.

This study addresses this gap by evaluating the impact of a standardized 21-day inpatient alcohol detoxification protocol on cardiovascular and hematological outcomes. In addition, it examines the relationship between changes in BMI, lipid profile, and Troponin-I and systemic oxidative stress markers—namely thiobarbituric acid reactive substances (TBARS), glutathione peroxidase, and catalase—to delineate patterns of early biological recovery. Specifically, the study aims to identify which physiological systems demonstrate rapid stabilization and which exhibit persistent dysfunction following short-term abstinence.

## Subjects and methods

### Study design and clinical setting

A prospective, observational, before-and-after cohort study was conducted between March 31, 2024, and December 31, 2024, at Al Amal Mental Health Hospital in the Kingdom of Saudi Arabia. The study was designed to evaluate the physiological and biochemical changes associated with acute alcohol cessation and early recovery among patients with severe Alcohol Use Disorder (AUD). Ethical approval for the study protocol was granted by the Regional Research Ethics Committee in Qassim Province under the Saudi Ministry of Health (Registration No. H-04-Q-001). The research employed a single-arm, self-controlled longitudinal design for the AUD cohort, in which each participant served as his own baseline control, allowing direct assessment of changes from active sub-acute alcohol toxicity to early abstinence stabilization. In addition, a healthy comparison group was included to provide baseline reference values for hematological and cardiovascular parameters. The study cohort consisted of 75 non-smoking male participants, including 50 hospitalized patients diagnosed with severe AUD and 25 healthy controls. To minimize confounding variables, the control group was matched to the AUD group with respect to age, dietary habits, and nutritional intake.

### Participant selection and eligibility criteria

All participants underwent detailed medical history documentation and structured psychiatric evaluation. Diagnostic assessments were conducted by an experienced psychiatrist according to the criteria outlined in the Diagnostic and Statistical Manual of Mental Disorders, Fifth Edition.

### Inclusion and exclusion criteria

To ensure group homogeneity, eliminate confounding systemic variables, and maximize the internal validity of the findings, strict inclusion and exclusion criteria were rigorously applied. Eligible participants for the alcohol use disorder (AUD) cohort were adult males aged 18–60 years with a documented history of continuous alcohol dependence for at least one year who were admitted voluntarily for a standardized 21-day inpatient detoxification protocol. Healthy controls were recruited from the same socio-demographic region and matched for age and general dietary habits. Notably, all participants in both the AUD and healthy control cohorts were verified non-smokers, effectively eliminating nicotine consumption and its associated systemic inflammation as a confounding variable. Conversely, individuals were excluded from the study if they presented with established chronic medical comorbidities that could independently alter cardiovascular, lipidemic, or oxidative stress biomarkers; these included pre-existing hypertension, diabetes mellitus, severe renal impairment, advanced hepatic disease (such as decompensated cirrhosis), or hematological and immunological disorders. To isolate the direct metabolic effects of alcohol cessation, participants using lipid-lowering therapies (e.g., statins, fibrates), anti-inflammatory drugs, or regular antioxidant/mineral supplements (including Vitamins C, E, or zinc) within 30 days prior to enrollment were excluded. Additional exclusion criteria comprised acute clinical instability, newly diagnosed acute illnesses during screening, severe manifestations of Alcohol Withdrawal Syndrome (AWS) requiring emergency intensive care intervention (such as delirium tremens or withdrawal seizures), an alcohol dependence duration of less than one year, or falling outside the specified age range. Female patients were also excluded from the current protocol to maintain structural cohort uniformity based on institutional admission epidemiology.

### Therapeutic intervention protocol

Upon admission, all eligible AUD participants were enrolled in a standardized, supervised 21-day inpatient detoxification and stabilization program. To ensure treatment consistency and minimize therapeutic confounding, all participants received the same medical management protocol within a controlled inpatient setting. The acute withdrawal phase (Days 1–10) was managed using a symptom-triggered, tapered benzodiazepine regimen consisting primarily of Diazepam to control autonomic hyperactivity and prevent withdrawal-related seizures. For craving reduction and maintenance stabilization throughout the inpatient period, Gabapentin was administered according to institutional treatment guidelines. To reduce the risk of neurological complications, including Wernicke–Korsakoff syndrome, participants received high-dose parenteral Thiamine (Vitamin B1; 500 mg/day) during the initial five days of admission, followed by oral vitamin and multivitamin supplementation. Nutritional rehabilitation was further supported through a standardized hospital diet providing approximately 2,500 kcal/day to address chronic nutritional deficiencies and systemic oxidative stress commonly associated with prolonged alcohol misuse. Participants remained under continuous inpatient supervision throughout the 21-day period. Abstinence from alcohol was strictly enforced within the hospital environment and verified through serial urinary alcohol testing in addition to daily clinical monitoring.

### Data collection timeline and measurement framework

The study measurement framework was divided into two predefined time points to capture the transition from active alcohol toxicity to early physiological recovery. Baseline assessments, designated as time point zero ($T_{\text{0}}$), were conducted within the first 24 hours of hospital admission before the initiation of full detoxification therapy. At this stage, anthropometric measurements including Body Mass Index (BMI) were recorded, and fasting venous blood samples were collected for evaluation of lipid profile parameters, cardiac biomarkers, and complete blood count indices. Follow-up assessments, designated as time point one ($T_{\text{1}}$), were performed on Day 21 immediately prior to patient discharge. The same clinical evaluations and laboratory investigations conducted at baseline were repeated at $T_{\text{1}}$, enabling direct longitudinal comparison of hematological and cardiovascular parameters before and after supervised alcohol abstinence and detoxification.

### Personal data

A variety of personal and medical data for every participant were collected. This information included their age, sex, and individual habits, as well as their toxicological history (e.g., substance use) and any record of medical diseases or drug treatments. A complete physical examination was also performed and documented. Finally, the Body Mass Index (BMI) in kg/m^2^ was calculated based on each participant's measured height and weight.

### Blood Sample Collection and Storage

Blood samples were collected from each participant and control subject using a sterile, disposable syringe via clean venipuncture under aseptic conditions. 10 ml of blood was drawn from every individual. The collected blood was processed, and the serum samples were transferred into tightly sealed vials. These vials were immediately stored at −80 ◦C to preserve them until the laboratory analysis could be performed [12]. The blood samples were critically collected at two stages: upon admission to the hospital before treatment began, and again after the detoxification period was completed.

### Determination of urinary alcohol level

The concentration of alcohol in urine was figured out using an automated technique combining ultra-micro distillation with an alcohol dehydrogenase enzymatic reaction. Adapted for a Technicon AutoAnalyser, the process involves distilling ethanol from the sample matrix followed by an enzymatic reaction where ethanol is oxidized by ADH and NAD+ to produce NADH, which is then quantified spectrophotometrically at 340 nm [13].

### Determination of Complete blood counts

An automated Celltac MEK-6410K haematology cell counter (manufactured by Nihon Kohden Corporation in Tokyo, Japan) was utilized to precisely measure a standard set of parameters relevant to blood health. These measurements included the haematocrit (HCT), haemoglobin (Hb) level, mean corpuscular volume (MCV), platelet count (PLT), red blood cell (RBC) count, and white blood cell (WBC) count, providing a detailed profile of the cellular components of the participants’ blood. [14].

### Determination of troponin

Cardiac Troponin-I (cTnI) was quantified in human serum utilizing a commercial high-sensitivity Troponin-I (hs-cTnI) ELISA Assay Kit (Eagle Biosciences, Inc., Nashua, NH, USA). This assay employs an immunoenzymatic colorimetric method for the quantitative determination of cTnI, following the established reference protocol described by Apple et al. [15]. The minimum detectable concentration (sensitivity) of the assay is 0.01 ng/mL.

### Determination of serum lipid profile

Serum total cholesterol was measured out according to the method of Allain et al. (1974) using Kits of, Linear Chemicals, S.L (Spain) [16]. Serum triglycerides were measured out according to the method of Fossati and Principle (1982) using Kits of, Linear Chemicals, S.L (Spain) [17]. The method for measurement of serum HDL-cholesterol was determined according to the method of Burstein et al. (1970) and Lopes-Virella et al. (1977) using Kits of, Linear Chemicals, S.L (Spain) [18,19]. Low density lipoprotein cholesterol (LDLc) and very low-density lipoprotein cholesterol (VLDLc) was calculated according to Friedewald et al., (1972) [20].

### Determination of oxidative stress markers

Thiobarbituric Acid-Reactive Substances (TBARS): TBARS, an indicator of lipid peroxidation (damage to cell membranes), were measured in the serum using the method described by Esterbauer and Cheeseman (1990) [21]. **Catalase (CAT) Enzyme Activity:** The activity of the Catalase enzyme (EC1.11.1.6), which breaks down harmful hydrogen peroxide, was measured in tissues using the technique outlined by Aebi (1984) [22]. **Glutathione Peroxidase (GPx) Levels:** The levels of the antioxidant enzyme GPx were determined in the blood using the Paglia and Valentine method. For this, a Commercial Biochemical assay kit (RANSEL; Glutathione Peroxidase kit by RANDOX) was employed [23].

### Statistical analysis

All collected data were organized into tables and analyzed using the IBM SPSS Statistics for Windows software program (version 27, IBM Corp., Armonk, N.Y., USA). Categorical data (like types or groups) were summarized using numbers and percentages. For numerical data, a Shapiro-Wilk test was first conducted to check if the data were normally distributed. Numerical data that were found to be normally distributed were then reported using the mean ± standard deviation. Comparisons between Group I and Group II were done by the Independent Samples T-test. Alternatively, Paired T test was adopted for comparisons between subgroups of alcohol abuse patients before treatment (Group IIAA) and after treatment (Group IIAT). Furthermore, Pearson correlation was applied to investigate the relationships between each of age of patients, duration of addiction, and urinary alcohol levels and all the laboratory parameters studied both before and after therapy and data were presented as correlation coefficient r and p value. P values <0.05 was considered statistically significant. A post-hoc power analysis was conducted using G*Power (version 3.1.9.7). For the longitudinal pre- versus post-detoxification comparisons within the AUD group (n=50), the study achieved a statistical power (1 - β) > 95% to detect medium-to-large effect sizes (d ≥ 0.55) at a significance level of α = 0.05.

### Ethics statement

This study was reviewed and officially approved by the Regional Research Ethics Committee in Qassim Province, which is registered with the National Committee of Bio & Med. Ethics (NCBE) under Registration No. H-04-Q-001, operating under the Saudi Ministry of Health. Institutional approval was also obtained from Qassim University. All procedures involving human participants strictly adhered to the ethical standards and regulations set by the NCBE. Prior to participation, informed written consent was obtained from all individual participants included in the study. Participants were explicitly informed of their right to withdraw from the study at any time without penalty. In the event of a participant's withdrawal, they were replaced by a new participant following the acquisition of their formal consent. To ensure data privacy and confidentiality, all participant data was fully anonymized and coded. The study findings will be disseminated and published in compliance with international research publication policies.

## Results

The present study is a prospective observational before–after cohort study conducted on a sample of 50 male patients with alcohol use disorder admitted to Al Amal Mental Health Hospital in the Qassim region of Saudi Arabia for detoxification, alongside a control group of 25 healthy male volunteers. All participants in both the alcohol abusers and control groups were confirmed non-smokers. To minimize confounding variables, the control group was selected to match the alcohol abusers’ group in terms of age, dietary habits, and nutritional intake. Throughout detoxification, eleven of the patients ceased to continue with their treatment and were thus excluded from the alcohol abuser group and replaced.

The participants in this study spanned an age range, from 18 to over 59 years, with mean ages of 35.6 ± 8.5 and 35.4 ± 10.60 year for control and alcohol abuse group, respectively. No significant difference between control group and alcohol abuse group occurred in terms of mean age (*p* = 0.814) (Table 1). Table 1 showed how most patients were aged between 26 and 45 years old. This group formed 72.00% and 68.00% of group I and group II, respectively.

**Table 1:** Assessment of the age (years) in the control and the before treatments alcohol abusers’ groups (n = 75).

Age groups	Group I (N = 25)		Mean ± SD	Group II (N = 50)		Mean ± SD	P-Value
**18-25**	3	12.0%	35.6 ± 8.5	7	14.0%	35.4 ± 10.6	0.814
**26-35**	11	44.0%		22	44.0%		
**36-45**	7	28.0%		12	24.0%		
**46-59**	4	16.0%		9	18.0%		

*Significant at p < 0.05. Group I: Control group, Group II: alcohol abuser before treatment.

The range of duration of abuse was 1.5 to 25 years, with a mean of 7.5 ± 6.1 year. The urinary alcohol level on admission range was 120–822 mg/dL, with a mean of 332.8 ± 129.3 mg/dL.

### Body mass index and vital signs

**Fig 1:**
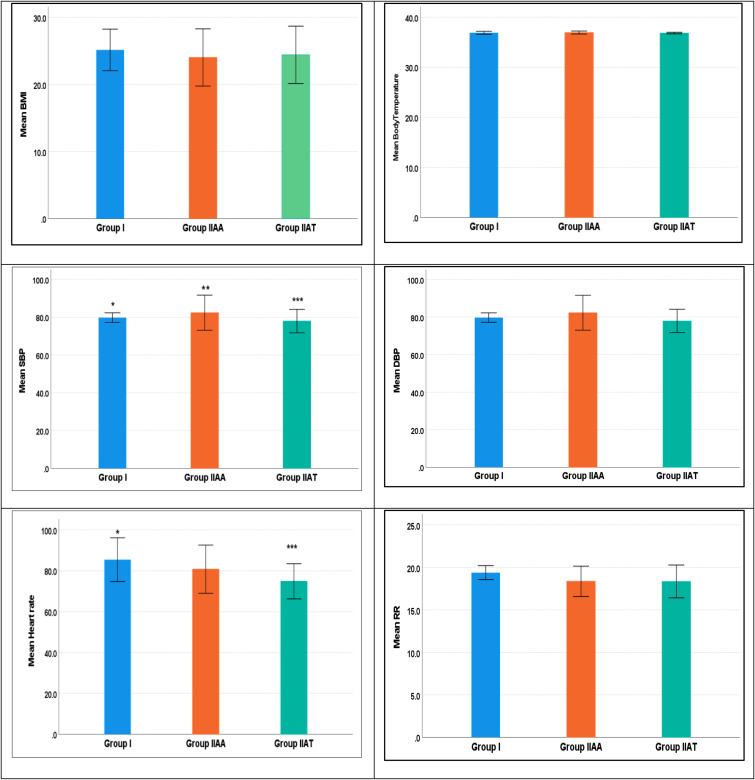
Changes in body mass index and vital signs before and after detoxification. Comparison of BMI, systolic and diastolic blood pressure, heart rate, temperature, and respiratory rate between control, pre-treatment (IIAA), and post-treatment (IIAT) groups. Data are presented as mean ± SD. *p < 0.05.

The study found that mean body mass index values was 25.1 ± 3.1 kg/m^2^ for control group. For groups IIAA and IIAT, the mean body mass index values were 24.0 ± 4.3 kg/m² and 24.4 ± 4.3 kg/m², respectively. Fig 1 showed no significant change in mean body mass index values for groups IIAA and IIAT compared to the control group (*p* = 0.123 and 0.223 respectively). Mean body mass index showed no significant diffrences in IIAT group when compared to IIAA group (*p* = 0.325).

The study found that the mean temperature values was 36.9 ± 2.8 °C for control group. The mean temperature values were 36.9 ± 0.3 and 36.9 ± 0.1°C for groups IIAA and IIAT, respectively. As per Fig 1 showed no significant differences in groups IIAA and IIAT when compared to control group (*p* = 0.250 and 0.193). There were no significant differences after receiving the treatment compared to IIAA group (*p* = 0.455).

The study found that the mean systolic blood pressure values was 127.4 ± 5.7 mmHg for the control group. The mean systolic blood pressure values were 137.6 ± 10.9 and 121.6 ± 7.0 mmHg for groups IIAA and IIAT, respectively. Fig 1 showed significant increase in mean systolic blood pressure values for IIAA group and significant decrease in IIAT group as compared to the control group (*p* = 0.002* and 0.003*). Mean systolic blood pressure showed a significant decrease in IIAT group as compared to IIAA group (*p* = < 0.001*).

The study found that mean diastolic blood pressure values was 79.8 ± 2.5 mmHg for control group. Mean diastolic blood pressure values were 82.3 ± 9.3 and 78.0 ± 6.2 mmHg for groups IIAA and IIAT, respectively. Fig 1 showed no significant difference in mean diastolic blood pressure values for groups IIAA and IIAT when compared to the control group (*p* = 0.089 and 0.301). Mean diastolic blood pressure showed no significant differences in IIAT group when compared to IIAA group(*p* = 0.086).

The study found that the mean heart rate values was 77.8 ± 6.8 bpm for control group. The mean heart rate values were 80.8 ± 11.8 and 74.9 ± 8.6 bpm for groups IIAA and IIAT, respectively. As per Fig 1, a significant increase was clear in the mean heart rate values IIAA group when compared with the control group (*p* < 0.001*) and no significant difference in the mean heart rate values for IIAT group when compared with the control group (*p* = 0.101). The mean heart rate value showed significant decrease IIAT group as compared to IIAA group (*p* = 0.003*).

The study found that the mean respiratory rate values was 19.6 ± 0.8 cpm for control group. The mean respiratory rate values were 18.4 ± 1.7 and 18.4 ± 1.9 cpm for groups IIAA and IIAT, respectively. As per Fig 1, no significant difrences in mean respitory rate values for groups IIAA and IIAT were discernable when compared with the control group (*p* = 0.435 and 0.167). The mean respiratory rate value showed no significant change in IIAT group compared with IIAA group (*p* = 0.138).

### Complete blood counts

Table 2 shows Assessment of the complete blood counts in the control and the alcohol abusers before and after the treatment. The study found that mean RBCs values was 5.6±0.3 for control group. The mean RBCs values were 5.0±0.7 and 5.6±0.7 for groups IIAA and IIAT, respectively. Table 2 showed significant decrease in mean RBCs values for IIAA group and no significant diffrences in mean RBCs values for IIAT group compared to the control group (*p* = <0.001* and 0.466 respectively). Mean RBCs showed significant increase in IIAT group when compared to IIAA group (*p* =<0.001*).

**Table 2:** Assessment of the complete blood count parameters in the control and the alcohol abusers’ groups (n = 75).

Variables		Groups			P-Value		
		Group I (N = 25)	Group II (N = 50)		GI vs GIIAA	GI vs G IIAT	GIIAA vs GIIAT
			Group IIAA	Group IIAT			
RBCs (x10^12^/L)	Mean± SD	5.6 ± 0.3	5.0 ± 0.7	5.6 ± 0.7	<0.001*	0.466	<0.001*
Hb levels (g/dL)	Mean± SD	15.3 ± 0.7	14.0 ± 1.5	15.1 ± 1.6	<0.001*	0.217	<0.001*
Hct (%)	Mean± SD	45.6 ± 2.5	42.1 ± 3.9	46.1 ± 5.3	<0.001*	0.351	<0.001*
MCV (fL)	Mean± SD	86.3 ± 3.5	95.1 ± 11.7	91.2 ± 9.4	<0.001*	0.388	<0.001*
Platelets (x10^9^/L)	Mean± SD	265.5 ± 49.0	251.6 ± 59.7	264.8 ± 40.2	0.159	0.474	0.099
WBCs (x10^9^/L)	Mean± SD	6.3 ± 0.8	8.4 ± 2.6	6.9 ± 2.0	0.002*	0.756	0.002*
Neutrophils (cells/μL)	Mean± SD	4.3 ± 0.8	3.2 ± 1.4	4.0 ± 1.4	0.003*	0.243	0.002*
Lymphocytes (cells/μL)	Mean± SD	1.9 ± 0.4	2.4 ± 0.9	2.7 ± 0.8	0.003*	<0.001*	0.042*
NLR	Mean± SD	2.3 ± 0.5	1.3 ± 0.7	1.8 ± 0.6	<0.001*	0.003*	<0.001*

*Significant at p < 0.05. Group I: Control group, Group IIAA: alcohol abuser before treatment, Group IIAT: alcohol abuser after treatment. SD: standard deviation. RBCs: red blood cells, Hb: hemoglobin, Hct: hematocrit, MCV: mean corpuscular volume, WBCs: white blood cells, NLR: neutrophil/ lymphocyte ratio

The study found that mean Hb values was 15.3 ± 0.7 for control group. The mean Hb values were14.0 ± 1.5 and 15.1 ± 1.6 for groups IIAA and IIAT, respectively. Table 2 showed significant decrease in mean Hb values for IIAA group and no significant diffrences in mean Hb values for IIAT group compared to the control group (*p* = < 0.001* and 0.217 respectively). Mean Hb showed significant increase in IIAT group when compared to IIAA group (*p* =<0.001*).

The study found that mean Hct values was 45.6 ± 2.5 for control group. The mean Hct values were 42.1 ± 3.9 and 46.1 ± 5.3 for groups IIAA and IIAT, respectively. Table 2 showed significant decrease in mean Hct values for IIAA group and no significant diffrences in mean Hct values for IIAT group compared to the control group (*p* = < 0.001* and 0.351 respectively). Mean Hct showed significant increase in IIAT group when compared to IIAA group (*p* =<0.001*).

The study found that the mean MCV value was 86.3 ± 3.5 fL for the control group. The mean MCV values were 95.1 ± 11.7 fL and 91.2 ± 9.4 fL for groups IIAA and IIAT, respectively. Table 2 showed a significant increase in mean MCV values for the IIAA group and no significant differences in mean MCV values for the IIAT group compared to the control group (p < 0.001 and p = 0.388, respectively). Mean MCV showed a significant decrease in the IIAT group when compared to the IIAA group (p < 0.001).

The study found that mean platelets values was 265.5 ± 49.0 for control group. The mean platelets values were 251.6 ± 59.7 and 264.8 ± 40.2 for groups IIAA and IIAT, respectively. Table 2 showed no significant change in mean platelets values for groups IIAA and IIAT compared to the control group (*p* = 0.159 and 0.474 respectively). Mean platelets showed no significant difrences in IIAT group when compared to IIAA group (*p* = 0.099).

The study found that mean WBCs values was 6.3 ± 0.8 for control group. The mean WBCs values were 8.4 ± 2.6 and 6.9 ± 2.0 for groups IIAA and IIAT, respectively. Table 2 showed significant increase in mean WBCs values for IIAA group and no significant diffrences in mean WBCs values for IIAT group compared to the control group (*p* = 0.002* and 0.756 respectively). Mean WBCs showed significant reduction in IIAT group when compared to IIAA group (*p* = 0.002*).

The study found that mean neutrophils values was 4.3 ± 0.8 for control group. The mean Neutrophils values were 3.2 ± 1.4 and 4.0 ± 1.4 for groups IIAA and IIAT, respectively. Table 2 showed significant decrease in mean neutrophils values for IIAA group and no significant diffrences in mean neutrophils values for IIAT group compared to the control group (*p* = 0.003* and 0.243 respectively). Mean neutrophils showed significant increase in IIAT group when compared to IIAA group (*p* = 0.002*).

The study found that mean lymphocytes values was 1.9 ± 0.4 for control group. The mean lymphocytes values were 2.4 ± 0.9 and 2.7 ± 0.8 for groups IIAA and IIAT, respectively. Table 2 showed significant increase in mean lymphocytes values for groups IIAA and IIAT compared to the control group (*p* = 0.003* and <0.001* respectively). Mean lymphocytes showed significant increase in IIAT group when compared to IIAA group (*p* = 0.042*).

The study found that mean NLR values was 2.3 ± 0.5 for control group. The mean NLR values were 1.3 ± 0.7 and 1.8 ± 0.6 for groups IIAA and IIAT, respectively. Table 2 showed significant decrease in mean NLR values for IIAA group and after treatment in mean NLR values for groups IIAA and IIAT compared to the control group (*p* < 0.001* and 0.003* respectively). Mean NLR showed significant increase in IIAT group when compared to IIAA group (*p* < 0.001*).

### Cardiac biomarker

**Fig 2:**
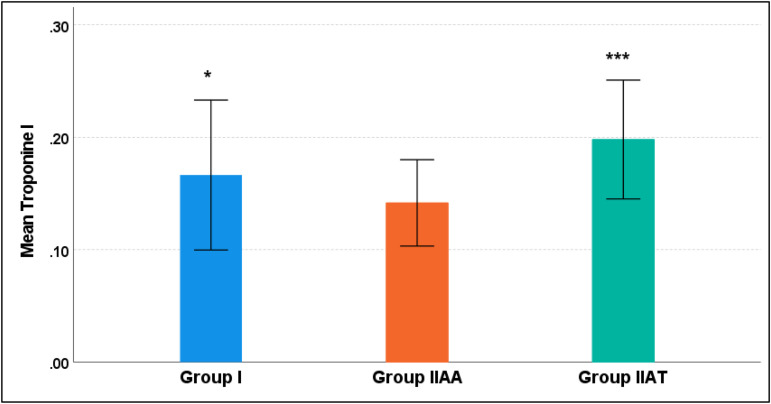
Changes in cardiac biomarker (Troponin-I) before and after detoxification. Serum troponin-I levels in control, pre-treatment (IIAA), and post-treatment (IIAT) groups. Significant elevation was observed in IIAA with reduction after treatment.

This study showed that, the mean troponin -I values was 0.13 ± 0.05 ng/mL for control group. The mean troponin -I values were 0.20 ± 0.05 and 0.14 ± 0.04 ng/mL for groups IIAA and IIAT, respectively. Fig 2 showed significant increase in mean troponin -I values for IIAA group and no significant differences after treatment after compared to the control group (*p* = < 0.001* and 0.092 respectively). Mean troponin -I showed significant decrease in IIAT group when compared to IIAA group (*p* = < 0.001*).

### Lipid profile

**Fig 3:**
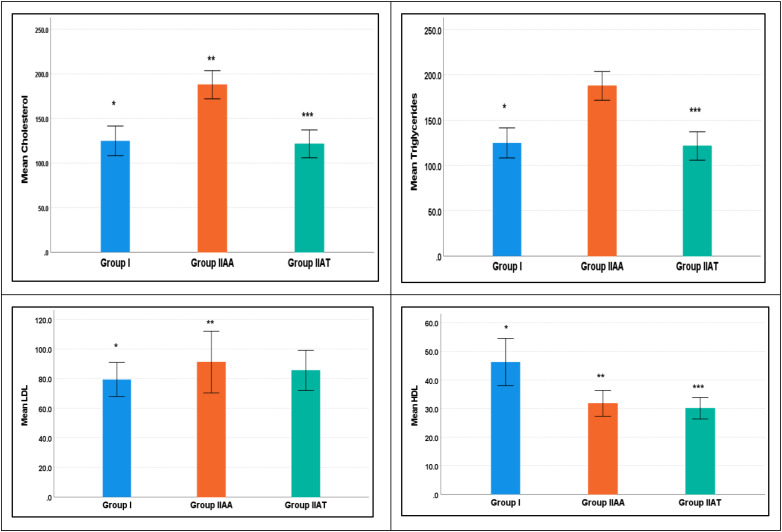
Lipid profile alterations following alcohol detoxification. Comparison of total cholesterol, triglycerides, LDL, and HDL across study groups. Detoxification resulted in partial normalization of lipid parameters.

The present study showed that, the mean cholesterol values was 134.4 ± 24.6 mg/dL for control group. The mean cholesterol values were 252.5 ± 30.0 and 223.8 ± 29.1 mg/dL for groups IIAA and IIAT, respectively. Fig 3 showed significant increase in mean cholesterol values for groups IIAA and IIAT compared to the control group (*p* = < 0.001* and <0.001* respectively). Mean cholesterol showed significant decrease in IIAT group when compared to IIAA group (*p* =<0.001*).

This study found that mean triglycerides values was 124.8 ± 16.6 mg/dL for control group. The mean triglycerides values were 187.7 ± 15.8 and 121.4 ± 15.6 mg/dL for groups IIAA and IIAT, respectively. Fig 3 showed significant increase in mean triglycerides values for IIAA group and no significant diffrences in IIAT group compared to the control group (*p* = < 0.001* and 0.195 respectively). Mean triglycerides showed significant decrease in IIAT group when compared to IIAA group (*p* =<0.001*).

The study reported that mean LDL values was 79.4 ± 11.5 mg/dL for control group. The mean LDL values were 91.2 ± 20.9 and 85.6 ± 13.6 mg/dL for groups IIAA and IIAT, respectively. Fig 3 showed significant increase in mean LDL values for groups IIAA and IIAT compared to the control group (*p* = 0.010* and 0.044* respectively). Mean LDL showed no significant difrences in IIAT group when compared to IIAA group (*p* = 0.115).

The present study showed that, the mean HDL values was 46.3 ± 8.3 for control group. The mean HDL values were 30.1 ± 3.8 and 31.8 ± 4.6 for groups IIAA and IIAT, respectively. Fig 3 showed significant decrease in mean HDL values for groups IIAA and IIAT compared to the control group (*p* = < 0.001* and <0.001* respectively). Mean HDL showed significant increase in IIAT group when compared to IIAA group (*p* = 0.022*).

### Oxidative stress biomarkers

**Fig 4:**
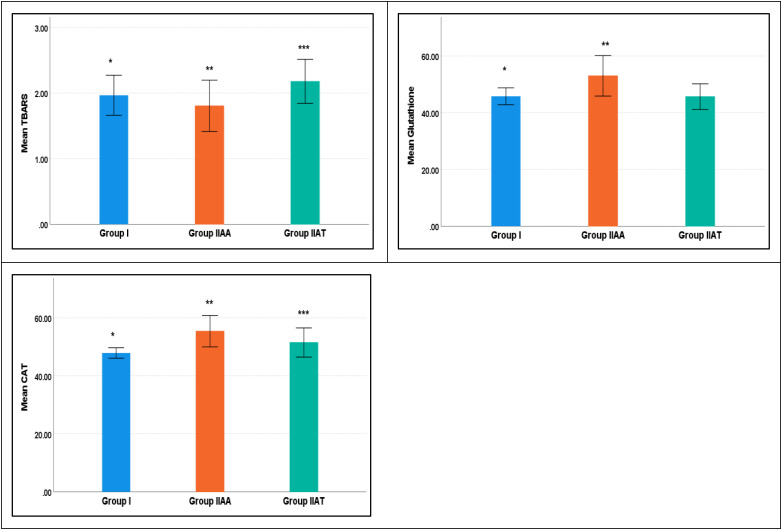
Oxidative stress biomarkers before and after detoxification. Levels of TBARS, glutathione peroxidase (GPx), and catalase (CAT) across groups. Persistent oxidative imbalance observed despite detoxification.

The present study showed that, the mean TBARS values was 2.0 ± 0.3 nmol/ml for control group. The mean TBARS values were 2.9 ± 0.5 and 2.6 ± 0.5 nmol/ml for groups IIAA and IIAT, respectively. Fig 4 showed significant increase in mean TBARS values for groups IIAA and IIAT compared to the control group (*p* = < 0.001* and <0.001* respectively). Mean TBARS showed significant decrease in IIAT group when compared to IIAA group (*p* = 0.002*).

Fig 4 showed that, the mean glutathione peroxide values was 45.8 ± 3.0 U/L for control group. The mean glutathione peroxide values were 39.6 ± 8.2 and 41.6 ± 6.8 U/L for groups IIAA and IIAT, respectively. The present study showed significant decrease in mean glutathione peroxide values for groups IIAA and IIAT compared to the control group (*p* = 0.002* and 0.002* respectively). Mean glutathione peroxide showed no significant change in IIAT group when compared to IIAA group (*p* = 0.087).

The present study showed that, the mean CAT values was 47.9 ± 1.8 U/L for control group. The mean CAT values were 63.8 ± 8.8 and 51.5 ± 5.0 U/L for groups IIAA and IIAT, respectively. Fig 4 showed significant increase in mean CAT values for groups IIAA and IIAT compared to the control group (*p* = < 0.001* and <0.001* respectively). Mean CAT showed significant decrease in IIAT group when compared to IIAA group (*p* =<0.001*).

### Correlations

Table 3 proves a significant weak positive correlation between BMI (r = 0.441, *p* = 0.001*), SBP (r = 0.312, *p* = 0.027*) and DBP (r = 0.386, *p* = 0.006*) measured before treatment and age of the patients. Likewise, there was a significant weak positive correlation between each of BMI (r = 0.387, *p* = 0.005*) and DBP (r = 0.351, *p* = 0.012*) and the duration of addiction. Alternatively, there was a significant negative weak correlation between RR (r = −0.424, *p* = 0.002*) counted before treatment and the urinary alcohol level obtained on admission. Other vital signs reached on admission did not show significant correlations with either age, duration of addiction, or the urinary alcohol level (All *p* values >0.05).

**Table 3:** Correlations between BMI and vital signs measured before treatment and age, duration of addiction, and urinary alcohol level (n = 50).

Before treatment	Age, years		Duration of addiction		Urinary Alcohol Level on admission	
	r	p-value	r	p-value	r	p-value
BMI (kg/m2)	**0.441**	0.001*	**0.387**	0.005*	−0.166	0.251
Temp. (C)	0.062	0.668	0.144	0.320	−0.203	0.158
SBP (mmHg)	**0.312**	0.027*	0.278	0.051	−0.197	0.171
DBP (mmHg)	**0.386**	0.006*	**0.351**	0.012*	−0.127	0.381
HR (bpm)	−0.058	0.691	−0.140	0.332	−0.063	0.666
RR (cpm)	−0.116	0.420	−0.116	0.432	**−0.424**	0.002*

*Significant at p < 0.05.r: Pearson correlation coefficient

Table 4 reveals a significant weak positive correlation between MCV and age (r = 0.329, *p* = 0.020*) as well as duration of addiction (r = 0.284, *p* = 0.046*), while RBCs showed a significant weak negative correlation with the age of the patients (r = **−**0.322, *p* = 0.022*). Otherwise, there were no significant correlations between CBC parameters got on admission and age, duration of addiction, or the urinary alcohol level (All p values >0.05).

**Table 4:** Correlations between complete blood counts obtained before treatment and age, duration of addiction, and urinary alcohol level (n = 50).

Before treatment	Age, years		Duration of addiction		Urinary Alcohol Level on admission	
	r	p-value	r	p-value	r	p-value
RBCs(10*12/L)	**−0.322**	0.022*	−0.246	0.085	−0.013	0.928
Hb levels(g/dL)	0.022	0.881	0.067	0.642	0.065	0.654
Hct (%)	−0.113	0.436	−0.020	0.890	−0.009	0.948
MCV (fL)	**0.329**	0.020*	**0.284**	0.046*	−0.023	0.873
Platelets (10*9/L)	−0.141	0.329	−0.209	0.145	0.112	0.440
WBCs (10*9/L)	0.059	0.682	0.046	0.752	−0.044	0.762
Neutrophils (cells/μL)	0.062	0.668	0.069	0.633	0.059	0.682
Lymphocytes (cells/μL)	0.023	0.872	−0.008	0.954	−0.259	0.070
NLR	0.151	0.295	0.140	0.333	0.185	0.189

*Significant at p < 0.05.r: Pearson correlation coefficient

Table 5 shows only a significant positive weak correlation between troponin -I level and the urinary alcohol level on admission (r = 0.309, *p* = 0.029*), however, the alcohol level showed a significant negative weak correlation with HDL (r = −0.380, *p* = 0.006*).

**Table 5:** Correlations between cardiac enzyme and lipid profile measured before treatment and age, duration of addiction, and urinary alcohol level (n = 50).

Before treatment	Age, years		Duration of addiction		Urinary Alcohol Level on admission	
	r	p-value	r	p-value	r	p-value
Troponin -I level (ng/mL)	0.052	0.719	−0.009	0.950	**0.309**	0.029*
Cholesterol (mg/dL)	−0.092	0.525	−0.066	0.651	−0.050	0.728
Triglycerides (mg/dL)	−0.029	0.840	−0.058	0.689	0.121	0.402
LDL (mg/dL)	0.068	0.924	0.172	0.231	−0.085	0.559
HDL (mg/dL)	−0.027	0.852	−0.119	0.411	−**0.380**	0.006*

*Significant at p < 0.05.r: Pearson correlation coefficient

The oxidative stress parameters including TBARS, Glutathione, and CAT did not show significant correlations with either age, duration of addiction, or the urinary alcohol level (All *p* values >0.05) as seen in Table 6.

**Table 6:** Correlations between oxidative stress parameters measured before treatment and age, duration of addiction, and urinary alcohol level (n = 50).

Before treatment	Age, years		Duration of addiction		Urinary Alcohol Level on admission	
	r	p-value	r	p-value	r	p-value
TBARS (nmol/ml)	0.267	0.060	0.188	0.192	0.179	0.213
Glutathione peroxide (U/L)	0.086	0.551	0.094	0.514	−0.055	0.703
CAT (U/L)	−0.174	0.227	−0.074	0.609	0.095	0.512

r: Pearson correlation coefficient

Concerning correlations after treatment of alcohol addiction, the following tables show the results.

Table 7 illustrates a significant positive weak correlation between BMI and each of age (r = 0.453, *p* < 0.001*), and duration of addiction (r = 0.377, *p* = 0.007*). The vital signs obtained after treatment did not have any significant correlations with either age, duration of addiction, or the urinary alcohol level (All *p* values >0.05).

**Table 7:** Correlations between BMI and vital signs measured after treatment and age, duration of addiction, and urinary alcohol level (n = 50).

After treatment	Age, years		Duration of addiction		Urinary Alcohol Level on admission	
	r	p-value	r	p-value	r	p-value
BMI (kg/m2)	**0.453**	<0.001*	**0.377**	0.007*	−0.232	0.106
Temp. (C)	−0.158	0.272	0.063	0.661	0.049	0.738
SBP (mmHg)	0.235	0.100	0.167	0.222	−0.197	0.170
DBP (mmHg)	0.205	0.154	0.232	0.105	−0.062	0.668
HR (bpm)	0.016	0.913	−0.087	0.546	0.060	0.681
RR (cpm)	0.145	0.312	0.074	0.608	−0.057	0.692

*Significant at p < 0.05.r: Pearson correlation coefficient

Table 8 reveals a significant weak positive correlation between MCV and age (r = 0.338, *p* = 0.016*) as well as duration of addiction (r = 0.283, *p* = 0.046*), and a significant weak positive correlation of hematocrit % with the level of alcohol (r = 0.470, *p* < 0.001*). The NLR showed a significant weak positive correlation with age (r = 0.308, *p* = 0.030*) as well as duration of addiction (r = 0.280, *p* = 0.049*). Otherwise, there were no significant correlations between CBC parameters got after completing treatment and age, duration of addiction, or the urinary alcohol level (All *p* values >0.05).

**Table 8:** Correlations between complete blood counts obtained after treatment and age, duration of addiction, and urinary alcohol level (n = 50).

After treatment	Age, years		Duration of addiction		Urinary Alcohol Level on admission	
	r	p-value	r	p-value	r	p-value
RBCs (x10^12^/L)	−0.269	0.059	−0.265	0.063	−0.105	0.469
Hb levels (g/dL)	0.002	0.922	0.009	0.949	−0.179	0.215
Hct (%)	0.081	0.575	0.015	0.917	**0.470**	<0.001*
MCV (fL)	**0.338**	0.016*	**0.283**	0.046*	−0.043	0.767
Platelets (x10^9^/L)	0.006	0.966	−0.067	0.643	0.144	0.317
WBCs (x10^9^/L)	0.003	0.982	−0.007	0.963	−0.142	0.324
Neutrophils (cells/μL)	0.029	0.842	0.046	0.750	−0.057	0.695
Lymphocytes (cells/μL)	−0.083	0.565	−0.097	0.504	−0.136	0.346
NLR	**0.308**	0.030*	**0.280**	0.049*	0.107	0.462

*Significant at p < 0.05.r: Pearson correlation coefficient

Table 9 shows only a significant positive weak correlation between HDL measured after treatment and urinary alcohol level (r = 0.358, *p* = 0.011*).

**Table 9:** Correlations between cardiac enzyme and lipid profile measured after treatment and age, duration of addiction, and urinary alcohol level (n = 50).

After treatment	Age, years		Duration of addiction		Urinary Alcohol Level on admission	
	r	p-value	r	p-value	r	p-value
Troponin -I level (ng/mL)	−0.171	0.235	−0.1888	0.190	−0.140	0.332
Cholesterol (mg/dL)	0.004	0.979	0.058	0.687	−0.119	0.410
Triglycerides (mg/dL)	0.172	0.231	0.078	0.590	−0.225	0.074
LDL (mg/dL)	0.056	0.698	0.096	0.506	−0.211	0.142
HDL (mg/dL)	−0.045	0.755	−0.023	0.875	**0.358**	0.011*

*Significant at p < 0.05.r: Pearson correlation coefficient

Table 10 Also clarifies absence of any significant correlation between oxidative stress parameters measured after treatment and age, duration of addiction, and the urinary alcohol levels (All *p* values >0.05).

**Table 10:** Correlations between oxidative stress parameters measured after treatment and age, duration of addiction, and urinary alcohol level.

After treatment	Age, years		Duration of addiction		Urinary Alcohol Level on admission	
	r	p-value	r	p-value	r	p-value
TBARS (nmol/ml)	0.243	0.089	0.262	0.066	0.021	0.882
Glutathione peroxide (U/L)	0.142	0.326	−0.002	0.992	−0.008	0.956
CAT (U/L)	−0.179	0.213	−0.066	0.649	0.076	0.601

r: Pearson correlation coefficient

## Discussion

Alcohol has a determined history in Saudi Arabia, but many people and organizations voice their objection to its existence in the Arab society, with several others being anxious because of the rising number of users and abusers every year. There is a scarceness of reliable information on the exact number of individuals who consume alcohol, but several commentators have posited a range of causes which have triggered the elevated usage of alcohol in Saudi Arabia [4].

These factors are very influential since the huge economic growth that had arisen in the 1980s, as this has prompted an elevation in individual traveling to other states in which alcohol is considered legal. Globalization (in mass media and information technology) which had appeared since the 1990s was also known as a factor, specifically due to the internet and elevated exposure to odd cultural traditions and norms with extraneous values. Moreover, an enormous number of non-Saudi employees have come into Saudi Arabia since the 1980s, which have constituted a clear form came from the proliferation of non-native films and satellite channels [5].

Such factors are all linked with the growth in economic status. Various sociological influencers have also triggered the levels of consumption of alcohol upwards. The age group most influenced by the sophistications of alcohol usage is the 26–45 group (68%). Alcoholic patients in one research ranged in age ranged from 20 to 36 years old, with the mean age range being 32–36 years [24]. An international survey has demonstrated that the misuse of alcohol among younger people is rising, with age groups influenced ranging from 18 to 54 years [25]. These outcomes are consistent with the result of present research.

The connection among alcohol abuse and Body Mass Index (BMI) has been the interest of several research because the consumption of alcohol can drive complicated influences on the body weight and composition. Chronic alcohol abuse can affect BMI in incalculable ways, relying on factors like, the quantity and frequency of alcohol consumption, the type of alcohol consumed, and the individual’s overall lifestyle [26].

Ghibaudi et al., 2002 demonstrated that the moderate consumption of alcohol was accompanied by an elevated BMI in males and a decreased BMI in females, predicting a gender-specific relationship. The research also proves that the consumption of alcohol was positively connected to abdominal obesity (higher waist-to-hip ratio), which is a threatening factor for cardiovascular illnesses. The study anticipated that alcohol could affect the body weight via elevated caloric intake (alcohol is energy-dense) and alterations in metabolism. Despite that, the influence was reliant on both the amount of alcohol consumed and the gender of the patient. Males had the highest potential to experience weight gain, whereas females, specifically those with moderate intake of alcohol, were more likely to experience declines in weight or lower BMI [27].

The way alcohol consumption influences body weight and the potential development of obesity differs between men and women due to distinct patterns of caloric intake. Specifically, males typically treat alcohol as an addition to their existing diet, contributing extra calories to their daily total energy intake [28]. In contrast, women often tend to use alcohol as a substitute for other calorie sources, such as reducing carbohydrate consumption, which may result in minimal or no overall increase in their total calorie ingestion [33]. These fundamental differences in how alcohol is integrated into the daily energetic balance must be considered when analyzing body weight outcomes, as the effect on weight gain is likely to vary significantly between the sexes.

Moreover, in another study, there are conflicts with the overall studies mentioned above, in which it was found that the consumption of chronic alcohol was connected to elevation in the visceral fat and decreased mass of muscle in the participating individuals, in addition to the absence of unambiguous change in the overall weight of body or BMI. This predicts that redistributing fat and waste of muscle could happen regardless of a realized alteration in the weight of body [29]. These outcomes are also aligned to the results of the current study.

The consumption of Alcohol can have a highly realized influence on the vital signs of the body, like, the heart rate, the pressure of blood, the rate of respiration, the temperature of body. The influences rely on several factors like, the quantity of consumed alcohol, the individual tolerance mechanisms, the severity of use and chronic patterns, besides the total health status of the individual. Whilst the moderate intake of alcohol may have mild influences, the heavy or chronic intake can result in more severe alterations in the vital signs of the body [30].

Alcohol can elevate the heart rate because of activation of the sympathetic nervous system (the “fight or flight” response), that is usually triggered by alcohol. Alcohol can make stimulation of the release of catecholamines (e.g., epinephrine and norepinephrine) which elevate the heart rate [31].

Earlier research explores the method by which chronic consumption of alcohol can influence the heart rate variability (HRV), that indicates the balance of the autonomic nervous system as well as the function of heart. It was proven that the abuse of alcohol results in diminished HRV, which can elevate the risk of arrhythmias, involving atrial fibrillation. Some research anticipate that the cardiac arrhythmia driven by alcohol may be tied to impairments in the autonomic control of the rate of heart [32]. The outcomes of this research demonstrated unprecedented elevation of heart rate levels for alcohol abuser group prior to treatment when holding a comparison with the control group (p < 0.001*), that is owed to normal values after treatment, that is relevant to the earlier studies.

Alcohol typically results in a temporary diminish in blood pressure. This is because of the vasodilation influence of alcohol, where there is an expansion of blood vessels, which makes a drop in the pressure of blood. Despite that, the influence is transient and may change relying on the quantity of consumed alcohol [33].

Chronic consumption of alcohol (specifically in enormous amounts) can have hypertensive influence on the long run (elevated blood pressure). Intensified drinking is a well-known reason of essential hypertension and is one of the pro-founding participators to elevate blood pressure. This influence is thought to arise from the impact of alcohol on the sympathetic nervous system, the renin-angiotensin-aldosterone system, and the balance of fluid. Even moderate drinking can lead to a cumulative influence on blood pressure as time passes. Individuals with alcohol use alterations may face sustained elevated blood pressure, which raises the threat of cardiovascular illnesses such as, the heart attacks and strokes [34].

Ezzati et al., 2002 shedded light onto that even moderate drinking can result into elevated blood pressure and predicted that minimising the consumption of alcohol can aid in controlling hypertension [35]. The outcomes and results of the current research are constant with such findings as demonstrated it had elevation in the mean systolic blood pressure levels for the group of the abusers of alcohol prior to treatment in comparison to the control group. Mean systolic blood pressure showed a realized decline in IIAT groupin comparison to the group of the alcohol abusers before treatment. These findings support the clinical recommendation for limiting consumption of alcohol as part of an overall strategy to manage hypertension and minimising cardiovascular threats.

Alcohol has a depressant influence on the central nervous system (CNS), that involves respiratory depression. In insignificant quantities, alcohol may lead to a slight slowing of the rate of respiration, but in elevated doses, it can significantly decline the respiratory drive, resulting in respiratory depression. Severe intoxication can lead to hypoventilation (slow and shallow breathing), and in extreme statuses, respiratory arrest may happen, specifically when combined with other depressant factors of the CNS (e.g., benzodiazepines or opioids) [36].

In individuals who abuse alcohol in a chronic manner, depressant influences may be more realized, and using alcohol in the long run can also take part in sleep apnea, where stops of breathing and starts during sleep occur because of relaxation of muscle with poor control of the airway [37]. The result of the current research demonstrated no significant differences in the mean respitory rate levels for alcohol abuser group prior to and after treatment were realized in comparison to the control group. These findings may be as a result of the alcoholic drink’s types and doses which were different from that utilized by individuals involved in those studies.

Alcohol leads to vasodilation, that can make individuals feel a warmer sensation because of the dilation of the blood vessels in the skin. Despite that, this influence can also result in loss of heat from the body, because heat is lost via the surface of the skin, specifically in cold environments. This is the reason that individuals may sense heat after drinking alcohol but are at high threat of hypothermia in cold statuses [38]. This earlier study found that consumption of ethanol results in altered thermoregulation, specifically by making vasodilation and elevated loss of heat. It suggests that alcohol makes disruptions of the body to sustain core temperature, elevating the threat of hypothermia in cold environments [9]. The result of our study demonstrated no significant differences between control group and groups IIAA and IIAT that mean the emergency treated take in course. The explanation of such findings lies in that the environment of Saudi Arabia is worm.

Both acute and chronic alcohol consumption negatively affect the body's blood system (hematological effects) through two primary pathways: direct toxicity and indirect metabolic disruption [39,40]. The direct influence involves alcohol damaging the blood-forming organs like the bone marrow, as well as being toxic to the precursors of blood cells and the mature Red Blood Cells (RBCs), White Blood Cells (WBCs), and platelets. This damage leads to a reduced number of these cells or impairs their function. The indirect impacts are caused by resulting metabolic and physiological problems, such as the development of liver disease and crucial nutritional deficiencies—most notably folate deficiency—which severely alter the normal formation and function of various blood cells [39].

The deficiency of folate plays a significant and clear role in the development and progression of Alcoholic Liver Disease (ALD) [41], with most ALD patients exhibiting this nutritional shortfall. Folate deficiency disrupts methionine metabolism within the liver, a process where a protective molecule, S-adenosylmethionine (SAM), works to prevent the advancement of ALD. Research using a micro-pig model of alcoholic liver injury demonstrated that abnormal methionine metabolism—specifically characterized by reduced levels of SAM and the antioxidant glutathione in the liver, alongside increased levels of damaging lipid oxidation products—is associated with the development of steatohepatitis (fatty liver inflammation). Since methionine metabolism is regulated by folate, the co-occurrence of folate deficiency and dysfunctional methionine metabolism is considered a major feature of ALD [42].

Alcohol-induced liver damage secondarily harms red blood cells (RBCs) and the body's hemostatic mechanisms (blood clotting). Both the direct and indirect consequences of alcohol on the blood system significantly increase the risk of serious medical issues for alcohol abusers. A prominent example is anemia, which results from both reduced RBC production and impaired RBC function and metabolism. This anemia manifests through symptoms like fatigue, shortness of breath, lightheadedness, reduced mental ability, and abnormal heartbeats [43]. The fact that these findings align with the results of the current study further underscores the clinical relevance of alcohol's impact on hematological and cardiac health.

Previous research on alcohol consumption and Complete Blood Counts (CBC) show both agreement and variation when compared to the current study's results [44]. Like other findings, the current study is consistent in showing an elevation in Mean Corpuscular Volume (MCV) and a reduction in Red Blood Cells (RBCs) and hemoglobin among alcoholics [44,45]. However, a major discrepancy involves the White Blood Cells (WBCs): while one earlier study reported a decrease, the current study found elevated WBCs [46]. Furthermore, while another comparative study reported decreased platelet counts, the current study found no significant difference in platelet counts among the alcohol abusers, highlighting specific hematological variations that warrant further investigation.

A study by Berad and Chand (2019) comparing hematological parameters between alcoholic and non-alcoholic subjects showed a pattern of blood cell reduction that aligns with the current study's findings: specifically, their results demonstrated that the mean Red Blood Cell (RBC) count was normal in non-alcoholics but was progressively decreased in moderate alcoholics and even more so in severe alcoholics. The study by Berad and Chand (2019) also observed a decline in the total White Blood Cell (WBC) count and the platelet count as the severity of alcohol consumption increased, mirroring the dose-dependent reduction seen in Red Blood Cells (RBCs). These findings, which indicate that alcohol negatively impacts the production or count of multiple blood cell types, were consistent with the results of the current study [47].

Chronic alcohol consumption can have complex and varying effects on the immune system, particularly on white blood cells (WBCs) and their subtypes, including neutrophils and lymphocytes. While chronic alcohol use is often associated with immune suppression, some studies report increased WBC count, decreased neutrophil count, and increased lymphocyte count in individuals who chronically consume alcohol. These changes may be related to alterations in the immune response, immune cell dynamics, and the impact of alcohol on the bone marrow and lymphatic system [48].

An earlier study found that chronic alcohol consumption led to increased total WBC count, but with a significant reduction in neutrophil count and a compensatory increase in lymphocyte count. This phenomenon was attributed to alcohol's influence on bone marrow function and immune cell turnover. Alcohol-induced immune dysregulation resulted in alterations in the production and differentiation of leukocytes, leading to an increase in circulating lymphocytes and a decrease in neutrophils [48].

Another earlier study had higher WBC counts in chronic alcohol users, but this increase was due to elevated lymphocyte counts. In contrast, neutrophil counts were found to be significantly lower in alcohol-dependent individuals, even when their total WBC count was elevated. This was linked to alcohol-induced bone marrow suppression, which impaired the production of neutrophils while promoting the proliferation of lymphocytes, possibly as a compensatory response to alcohol-related immune dysfunction [49]. These findings are constant with the present study.

Alcohol-associated liver disease (ALD) represents a growing public health concern with an increasing disease burden. The mechanism of liver injury caused by alcohol is closely linked to inflammation. In clinical assessment, the Neutrophil-to-Lymphocyte Ratio (NLR) has emerged as a novel biomarker for inflammation. The NLR is considered both cost-effective and effective as a prognostic tool—meaning it helps predict the likely outcome—across a variety of inflammatory conditions [50].

Chronic alcohol use leads to a decreased NLR, which may reflect a shift in immune function towards lymphocyte predominance and neutrophil suppression. The earlier study found that chronic alcohol consumption was associated with decreased neutrophil counts and altered lymphocyte distribution. This resulted in a decrease in NLR, which was thought to be due to impaired neutrophil function and the compensatory increase in certain lymphocyte subsets, particularly T-cells. The study noted that alcohol-induced immune suppression may contribute to this immune shift [51]. Another study suggested that chronic alcohol use leads to a lower NLR, possibly due to dysfunctional neutrophil responses and a compensatory increase in lymphocyte numbers [49]. These findings are constant with the present study results.

Chronic alcohol consumption is a principal risk factor for cardiovascular disease, with evidence indicating that heavy intake precipitates acute myocardial depression and significantly elevates the long-term risk of cerebrovascular accidents and secondary cardiomyopathy [52–55]. At the cellular level, alcoholic cardiomyopathy is driven by mitochondrial fragmentation and pronounced oxidative stress. The resulting accumulation of reactive oxygen species (ROS) leads to the deleterious oxidation of lipids, proteins, and DNA within myocytes, alongside apoptosis and the structural modification of contractile proteins [56].

Our statistical analysis revealed that Troponin-I (cTnI) levels were significantly elevated in the AUD group at admission (0.20 ± 0.05 ng/mL) and remained significantly higher than controls even after the 21-day detoxification period. While participants did not present with acute coronary syndrome (ACS), these levels may reflect subclinical myocardial stress or transient biomarker elevation and myocyte ‘leakage’ caused by the direct toxicity of ethanol and its primary metabolite, acetaldehyde. The elevation of cTnI observed here may serve as a biomarker of early-stage cardiac remodeling or transient myocardial strain induced by systemic oxidative stress.

Furthermore, the persistent elevation of cTnI post-detoxification, despite the relatively rapid normalization of hematological indices, suggests that cardiac recovery is a significantly more prolonged process than hematological stabilization. This finding establishes a critical link between systemic toxicity—manifested by shifting hematological indices—and targeted organ damage within the cardiovascular system. While cTnI is the gold standard for diagnosing myocardial infarction, its elevation in non-coronary conditions is well-documented [57]. Consistent with earlier research, our findings suggest that chronic alcoholism induces the release of this cardiac marker through subclinical myofibrillar degeneration rather than acute ischemia [58]. These results indicate that ethanol-induced cardiotoxicity persists well beyond the acute withdrawal phase, necessitating extended cardiovascular monitoring following detoxification.

Chronic alcohol consumption significantly disrupts adipose tissue metabolism. These disruptions include the inappropriate activation of lipolysis, impaired insulin-mediated glucose uptake by fat cells, and disturbances in the way adipokines (hormones secreted by fat tissue) are expressed and secreted. Collectively, these effects create a pro-inflammatory environment within the body. Furthermore, these metabolic and inflammatory changes contribute to the ectopic deposition of fat (fat accumulation outside of adipose tissue, particularly) within the liver, which is the mechanism behind the development of alcoholic fatty liver disease [59].

Alcohol significantly alters lipid metabolism, particularly by inducing *de novo* fatty acid synthesis while simultaneously inhibiting fatty acid oxidation within the liver. The most common observed result of this disruption is an increase in plasma triglyceride levels [60]. When alcohol is consumed alongside fatty foods, the combination has a synergistic effect in raising plasma triglyceride concentrations, especially in individuals with normal lipid levels. This synergy occurs because alcohol inhibits the lipolysis of lipoproteins that are derived from the intestine. The increase in blood fat after eating is directly related to the individual's baseline fasting triglyceride concentration [61]. These findings may explain the results of this study.

Alcohol consumption influences the liver to increase the secretion of Very Low-Density Lipoprotein (VLDL). This occurs primarily because alcohol inhibits the liver's oxidation of free fatty acids (i.e., prevents the burning of fat), which in turn promotes the synthesis of hepatic triglycerides and subsequent VLDL secretion. However, a serious consequence arises in individuals with a pre-existing primary lipid disorder: alcohol consumption can cause them to develop severe hypertriglyceridemia (Type V). In contrast, regular alcohol use is also observed to raise plasma levels of High-Density Lipoprotein (HDL). Population studies have confirmed a positive correlation between alcohol intake and increased plasma levels of both HDL and triglycerides. One of the leading protective mechanisms suggested for alcohol's effects is its ability to induce these favorable changes in plasma lipoproteins, specifically by increasing HDL and potentially modifying LDL [60–63]. These findings are consistent with the current study results.

The metabolism of alcohol is strongly linked to heightened oxidative stress [64], which is defined as an imbalance where the body's production of free radicals overwhelms its antioxidant system [65]. The toxic effects of alcohol are primarily mediated by this oxidative stress, causing damage through mechanisms like the induction of lipid peroxidation (damage to cell membranes), DNA adducts, and DNA strand breaks At the cellular level, mitochondria are the main source of these damaging Reactive Oxygen Species (ROS), and alcohol-induced stress impairs crucial antioxidant activities, specifically those of Superoxide Dismutase (SOD), Catalase (CAT), and Glutathione Peroxidases (GPX). Furthermore, the enzyme CYP2E1 generates additional ROS, which intensifies the oxidative damage, triggering lipid peroxidation, protein and DNA damage, and ultimately leading to cell death [66].

The enzyme Catalase (CAT) plays a critical role in the antioxidant system by inactivating Reactive Oxygen Species (ROS) and their by-products [67]. The effect of alcohol on this enzyme is duration-dependent: while a shorter period of consumption decreases CAT activity, chronic alcohol intake typically causes an increase in CAT enzyme activity. This is partially linked to the role of Aldehyde Dehydrogenase-2, which is crucial for removing toxic aldehydes like malondialdehyde, a product of oxidative damage [68]. Consistent with the effects of long-term exposure, the present study found a significant increase in catalase values among the alcohol abusers compared to the healthy control group, suggesting an ongoing compensatory response to heightened oxidative stress.

Oxidative stress is a central factor in the development and progression of Alcohol Use Disorder (AUD), as ROS induces cellular oxidative stress that can specifically damage neurons. The body relies on crucial endogenous antioxidants—including Superoxide Dismutase (SOD), Glutathione Peroxidase (GPX), and reduced glutathione—to eliminate these harmful ROS. This detoxification process involves a two-step mechanism: first, SOD inactivates the radical superoxide by converting it into hydrogen peroxide (H_2_O_2_); then, GPX utilizes glutathione as an electron donor for H_2_O_2_, effectively removing the ROS and protecting the cell [69].

Chronic alcohol exposure elevates the generation of ROS, depletes cellular antioxidant defenses, and intensifies oxidative stress across multiple tissues, particularly in the liver. This oxidative stress is central to understanding how ethanol contributes to liver damage [70]. Long-term alcohol consumption is also known to impair mitochondrial activity [71]. Damage to mitochondrial structure and function caused by ethanol can further boost ROS production and promote cellular toxicity. Mitochondria isolated from rats fed ethanol chronically shows increased levels of lipid-peroxidation products, as well as elevated production of superoxide, hydrogen peroxide, and hydroxyl radicals [72,73].

The current study demonstrated that MDA levels were markedly elevated in individuals with alcohol dependence upon admission, a finding that aligns with previous reports [74]. Earlier research has frequently compared MDA concentrations during active alcohol consumption with those measured after abstinence, typically showing high MDA levels during ongoing drinking that gradually decline over the course of sobriety [75]. Our study observed a similar pattern; however, within the timeframe of hospitalization, MDA did not show a significant reduction even as withdrawal symptoms improved. This suggests that rapid detoxification in alcohol-dependent patients may help restore the balance between pro-oxidant and antioxidant systems, but notable decreases in MDA appear only after accounting for liver function. Prior studies indicate that serum MDA concentrations rise with increasing severity of liver disease, and that liver injury is closely linked to lipid peroxidation. Therefore, the marked increase in MDA among alcoholic patients likely reflects underlying liver dysfunction, which contributes to variations in MDA levels [76].

Cells possess a sophisticated antioxidant network composed of enzymatic defenses—such as superoxide dismutases (SOD), catalase (CAT), glutathione peroxidases (GPXs), and thioredoxin (Trx)—alongside various small-molecule antioxidants. Together, these components help maintain oxidative balance [77]. This multifaceted system, which includes both internally produced and externally supplied antioxidants, protects cells from the damaging effects of reactive oxygen species (ROS). Enzymatic free-radical scavengers form the primary line of defense by eliminating superoxide anions and hydrogen peroxide before they can generate the highly reactive hydroxyl radicals [78].

Both glutathione peroxidase and catalase—the primary enzymes responsible for detoxifying H₂O₂—have been shown to participate in clearing externally derived H₂O₂. The susceptibility of catalase to H₂O₂ appears to depend on the activity level of GPx. When glutathione peroxidase is upregulated, it helps maintain catalase activity at a higher level even in the presence of H₂O₂. The present study found significant decline in GPx activity compared with control group, which was like the result of another study [78]. Some studies found no significant change in concentration of the enzyme in the alcohol group [79].

Catalase (CAT) typically has only a limited role in alcohol metabolism, but it provides protection by breakin down hydrogen peroxide and related reactive species. Studies have shown that long-term alcohol consumption can lead to a dose-dependent increase in CAT activity in rat plasma. Consistent with previous research [24], the current study found that CAT activity was elevated in individuals with alcoholism upon admission but declined following detoxification.

Oxidative stress markers did not show significant improvement within the short detoxification period, which may reflect the longer time required for redox system recovery following alcohol cessation.

## Conclusion

Short-term alcohol detoxification was associated with partial improvement in cardiovascular and hematological parameters among patients with alcohol use disorder. Lipid profile components and hematological indices showed measurable changes following treatment, whereas oxidative stress markers did not demonstrate significant improvement within the limited detoxification period. These findings suggest that while early physiological recovery may occur during detoxification, normalization of oxidative stress may require longer durations of abstinence. Given the observational design, short follow-up, and potential confounding factors, these results should be interpreted with caution. Further longitudinal studies with larger and more diverse populations are needed to confirm these findings and evaluate long-term outcomes.

### Limitations and strengths

While this study provides significant insight into the short-term physiological and biochemical recovery of patients with alcohol use disorder (AUD), several limitations must be acknowledged. First, the cohort was exclusively male, reflecting the specific patient demographics and admission profiles at the study site, which may limit the generalizability of these findings to female populations. Second, the single-center nature and relatively small sample size (n = 50 AUD, n = 25 controls) reflect institutional recruitment constraints during the study period; however, this single-center framework offered a critical methodological advantage by minimizing treatment heterogeneity and guaranteeing a highly standardized inpatient environment. Third, the prospective 21-day follow-up period was strictly restricted to the acute inpatient detoxification timeframe; because this observational framework lacks a long-term post-discharge window, these longitudinal adjustments should be interpreted as strong clinical associations rather than definitive long-term causal proof. Lastly, although patients with overt chronic liver disease were rigorously excluded, subclinical nutritional deficiencies—specifically folate and vitamin B12—were not explicitly quantified, which may have independently influenced baseline macrocytic indicators like mean corpuscular volume (MCV).

## Supporting information

S1 dataset Data. (XLSX)
